# Biogeography rather than association with cyanobacteria structures symbiotic microbial communities in the marine sponge *Petrosia ficiformis*

**DOI:** 10.3389/fmicb.2014.00529

**Published:** 2014-10-10

**Authors:** Ilia Burgsdorf, Patrick M. Erwin, Susanna López-Legentil, Carlo Cerrano, Markus Haber, Sammy Frenk, Laura Steindler

**Affiliations:** ^1^Department of Marine Biology, Leon H. Charney School of Marine Sciences, University of HaifaHaifa, Israel; ^2^Department of Biology and Marine Biology, Center for Marine Science, University of North Carolina WilmingtonWilmington, NC, USA; ^3^Department of Life and Environmental Sciences (DiSVA), Polytechnic University of MarcheAncona, Italy; ^4^Department of Soil, Water, and Environmental Sciences, Agricultural Research Organization – Volcani CenterBet-Dagan, Israel; ^5^Department of Plant Pathology and Microbiology, Robert H. Smith Faculty of Agriculture, Food and Environment, The Hebrew University of JerusalemRehovot, Israel

**Keywords:** *Petrosia ficiformis*, sponge-microbe symbiosis, biogeography, cyanobacteria, *Synechococcus feldmannii*, 454 amplicon pyrosequencing, microbial diversity, porifera

## Abstract

The sponge *Petrosia ficiformis* is ubiquitous in the Mediterranean Sea and Eastern Atlantic Ocean, hosting a diverse assemblage of bacteria, including, in illuminated sites, cyanobacteria. Two closely related sponge color morphs have been described, one inside caves and at their entrance (white/pink), and one on the rocky cliffs (violet). The presence of the different morphs and their ubiquity in the Mediterranean (from North-West to South-East) provides an opportunity to examine which factors mostly affect the associated microbial communities in this species: (i) presence of phototrophic symbionts or (ii) biogeography. 16S rRNA gene tag pyrosequencing data of the microbial communities revealed that Chloroflexi, Gammaproteobacteria, and Acidobacteria dominated the bacterial communities of all sponges analyzed. Chlorophyll *a* content, TEM observations and DNA sequence data confirmed the presence of the cyanobacterium *Synechococcus feldmannii* in violet and pink morphs of *P. ficiformis* and their absence in white color morphs. Rather than cyanobacterial symbionts (i.e., color morphs) accounting for variability in microbial symbiont communities, a biogeographic trend was observed between *P. ficiformis* collected in Israel and Italy. Analyses of partial 18S rRNA and mitochondrial cytochrome c oxidase subunit I (COX1) gene sequences revealed consistent genetic divergence between the violet and pink-white morphotypes of *P. ficiformis*. Overall, data indicated that microbial symbiont communities were more similar in genetically distinct *P. ficiformis* from the same location, than genetically similar *P. ficiformis* from distant locations.

## Introduction

**Figure 1:**
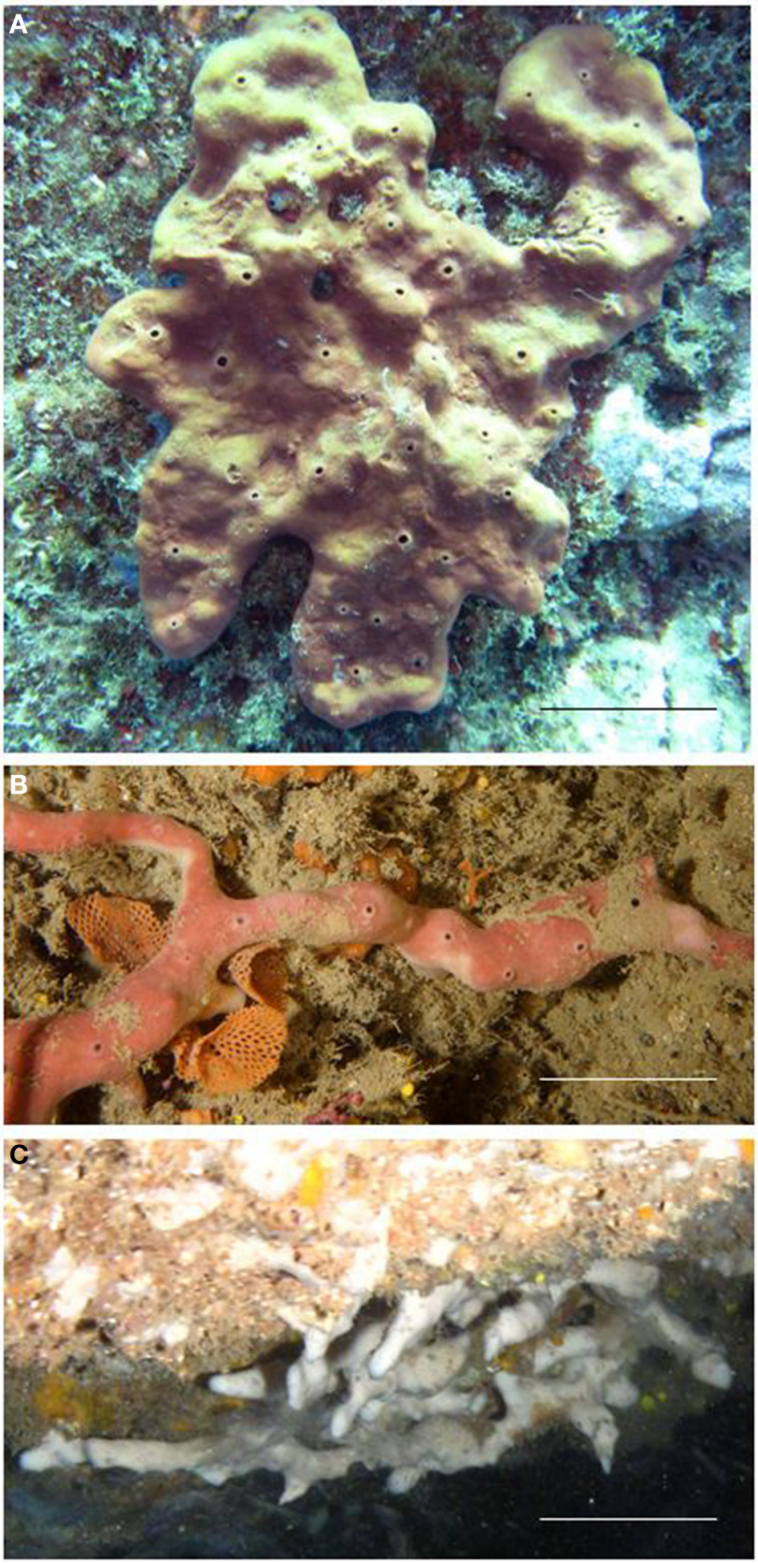
**Specimens of *P. ficiformis* showing the three morphs: (A) the massive violet type living in lighted areas (B,C) the creeping type with the pink and white color living in dim-light and dark condition, respectively**. Scale bars: **(A)** 5 cm, **(B,C)** 2 cm.

The marine demosponge *Petrosia ficiformis* (Poiret, 1789) is a sponge species found across the Mediterranean and in the Eastern Atlantic (Guo et al., 1998). It has been the focus of diverse studies that investigated: (i) the chemistry of the sponge and its associated microorganisms (Seidel et al., 1986; Bringmann et al., 2004; Lopez-Gresa et al., 2009; Pagliara and Caroppo, 2011), (ii) the ability to produce primmorphs (e.g., Mussino et al., 2013; Pozzolini et al., 2014), (iii) the identity of cyanobacterial symbionts (Usher et al., 2004; Steindler et al., 2005), and (iv) the molecular mechanisms underlying the interaction between sponge host and cyanobacteria (Arillo et al., 1993; Steindler et al., 2007). *P. ficiformis* has usually been described with two different morphs (Sarà et al., 1998): (i) a massive, violet-pigmented form, living in illuminated habitats harboring a dense population of intracellular cyanobacteria in the sponge cortex (the superficial layer of the sponge); and (ii) a slender pinkish or white morph, commonly found in shaded habitats (pink) and particularly in dark caves (white), where the sponges are free of phototrophic symbionts (Figure 1). *P. ficiformis* has been considered particularly suitable for studies on the establishment and maintenance of sponge-microbe symbiosis due to this facultative symbiosis with cyanobacteria (Steindler et al., 2007). *P. ficiformis* shows striking changes in its morphology in the presence and absence of cyanobacterial symbionts. These changes have been considered adaptive and relate to the size (light-exposed specimens are much larger than their dark cave counterparts), shape, surface skeleton, density of pores, and the metabolism (Vacelet and Donadey, 1977; Sarà et al., 1998). In the large and tabular specimens living on light-exposed cliffs, inhalant pores are very rare. Light-sheltered specimens have a lower symbiont concentration and the sponge becomes cylindrical in shape (Sarà et al., 1998). These changes affect the surface-to-volume ratio of the sponge whose values increase when chlorophyll *a* concentration decreases. In specimens with reduced cyanobacteria populations, a dense coat of vertical spicules is overlapped on the typical tangential spicular network. This arrangement could be tentatively interpreted as a light channeling system (Cattaneo-Vietti et al., 1996), likely improving light transfer in semi-dark habitats. In light-deprived specimens, the number of pores increases considerably, suggesting a more important role of the pumping system to support the feeding requirements. The aposymbiotic specimens (free of cyanobacteria) living in darkness can develop creeping branches or a spherical shape in case of low water movement, with an increased percentage of the surface covered by inhalant pores (Sarà et al., 1998). It has been shown that different morphs of *P. ficiformis* show differences in dehydrogenase activity and RNA content. It was proposed that in dark conditions *P. ficiformis* reacts to the absence of cyanobacteria by activating metabolic pathways able to maintain the cell reducing power.

*P. ficiformis* harbors a diverse and rich community of symbiotic bacteria (Schmitt et al., 2012a). The cyanobacterial symbiont, first described as *Aphanocapsa feldmannii* (Frémy in Feldmann, 1933) and later as *Synechococcus feldmannii* (Usher et al., 2004), is phylogenetically affiliated to free-living *Synechococcus*/*Prochlorococcus* species and not part of the widely distributed sponge-specific clade *Synechococcus spongiarum* (Steindler et al., 2005; Gao et al., 2014). The symbionts are absent from mature oocytes, spermatozoa, and embryos (Maldonado and Riesgo, 2009). Thus, microbes are likely acquired from ambient seawater by each new generation of juvenile sponges (Lepore et al., 1995; Maldonado and Riesgo, 2009). This differs from the vertical transmission of many sponge symbionts in other sponge species, including the transmission of the sponge-specific cyanobacterium clade *S*. *spongiarum* (e.g., Oren et al., 2005; Lee et al., 2009a; Webster et al., 2010; Gloeckner et al., 2013), and may also explain why most bacteria in the adult *P. ficiformis* sponge are found within bacteriocytes, as suggested in a more specific study on intergenerational transmission of microbial symbionts (Maldonado, 2007). Given the intracellular location (symbiosomes) of cyanobacterial symbionts in *P. ficiformis*, it is yet unknown how substrates are transferred from cortex to endosome (internal part of sponge), and whether certain heterotrophic bacteria within the sponge benefit from photosynthates and other substrates produced by cyanobacterial symbionts. The presence of cyanobacterial symbionts enhances the antioxidant defenses as compared to aposymbiotic specimens (Regoli et al., 2000) and may affect the heterotrophic bacterial community in such a way that different microbial communities could be expected in violet and white morphs of *P. ficiformis*.

The aim of this study was to determine which factors mostly affect the microbial community associated with *P. ficiformis*: presence of photosymbionts or biogeography. We compared the microbial communities of violet and white morphs of *P. ficiformis* from the northern Tyrrhenian Sea (Italy) and of violet morph in the north-western (Italy) and south-eastern (Israel) Mediterranean Sea. We took an additional violet sponge, and transferred half of it to a dark submarine cave, comparing the microbial communities of the violet and the “bleached” part (the half that lost its cyanobacterial symbionts after transfer to a cave). Finally, we amplified the 18S rRNA and cytochrome c oxidase subunit I (COX1) gene for triplicates of violet, pink (those growing at cave entrance), and white morphs of *P. ficiformis*.

## Methods

### Sponge collection

Three violet colored (PV1, PV2, PV3), two pink (PP1, PP3), and two white (PW2, PW3) *P. ficiformis* sponge samples were collected by SCUBA in August 2012 at depths of 10–13 m (violet), at the entrance (pink) and inside (white) of a submarine cave at 6 m in the Mediterranean Sea at Paraggi (GE), Italy (44°18′37.63″N; 9°12′48.01″E). An additional violet specimen (D), collected at 10 m depth, was cut into two halves. One half was transferred to the cave (Dt) and the other maintained at its original sampling location outside the cave. Dt was attached to the substrate inside the cave using an epossidic resin commonly used in aquariology. After almost 6 months (February, 2013), samples from these two sponge-halves (D, Dt) and additional white (PW1) and pink (PP2) *P. ficiformis* specimens were collected from the same cave. In May 2013, three violet colored *P. ficiformis* (termed here 106, 108, 111) were collected at a depth of approximately 20 m in Achziv on the Israeli Mediterranean coast (33° 2′44.19″*N*; 35° 5′7.58″E). After collection, all samples were immediately transferred to 100% ethanol and stored at −20°C until molecular analysis.

### DNA extraction, denaturing gradient gel electrophoresis (DGGE) and 454 amplicon pyrosequencing

DNA was extracted from all sponge samples using the Power Soil DNA Isolation kit, MoBio Laboratories (Carlsbad, CA) according to the manufacturer's protocol. Cortex and endosome parts of samples 106, 108, and 111 were separated first and extracted separately. Initial comparisons of microbial communities in the cortex and endosome of these sponges were conducted using triplicate PCR reactions with a universal primer set for bacteria (341F-GC and 907R, Muyzer et al., 1993) followed by DGGE fingerprinting, as previously described (Green et al., 2004). For subsequent analyses (454 gene tag pyrosequencing), samples 106, 108, and 111 represent equal concentration mixtures of DNA derived from cortex and endosome. Cortex and endosome of sample 106 were also sequenced separately. Extracted DNA samples were delivered for pyrosequencing to Molecular Research LP (Shallowater, TX, USA). The V6–V8 hypervariable region of the 16S rRNA gene was amplified by PCR using the HotStarTaq Plus Master Mix Kit (Qiagen, Valencia, CA) and primers 926f and 1392r (Engelbrektson et al., 2010) under the following conditions: 94°C for 3 min; followed by 28 cycles of: 94°C for 30 s, 53°C for 40 s, and 72°C for 1 min; and a final elongation step at 72°C for 5 min. All amplicon products were purified using Agencourt Ampure beads (Agencourt Bioscience Corporation, MA, USA). Samples were sequenced utilizing Roche 454 FLX titanium instruments following manufacturer's guidelines.

### Quality control and OTU clustering of 454 sequence data

Raw sequence data were processed using MOTHUR 1.33.0 (Schloss et al., 2009). The sequences were first trimmed of primer and tag sequences and de-noised by removing reads with low quality scores (using “average window,” average quality >25, window size = 50 bp, maximum homopolymer number per sequence = 8 and length >200 bp). The remaining sequences were aligned using a reference database (Silva database). Pyrosequencing errors were diagnosed using a pseudo-single linkage algorithm (pre.cluster command). Chimera sequences were detected and removed from the dataset using UChime (http://www.drive5.com/uchime/). All sequences were classified according to RDP (PDS) version 9 and mitochondrial, eukaryotic and unknown sequences were removed. For the remaining sequences, a pairwise distance matrix between aligned sequences was calculated and the sequences were clustered into operational taxonomic units (OTUs) based on 97% sequence identity (average neighbor algorithm). OTUs were classified according to both RDP version 9 and SILVA version 102. Ambiguous results were classified by manual BLASTn (Altschul et al., 1990) against SILVA 115 non-redundant database and a web based BLAST tool (http://blast.ncbi.nlm.nih.gov/). SILVA 115 non-redundant database was downloaded from the SILVA official web site (http://www.arb-silva.de).

### Statistical analyses of microbial diversity and community structure

DGGE images were analyzed with the Fingerprinting II® software (Bio-Rad Laboratories, Hercules, CA, USA), with cluster analyses calculated using unweighed pair group methods with arithmetic means (UPGMA) based on a Pearson r distance matrix. Significant differences in microbial community similarity between the cortex and endosome tissues were determined using permutational multivariate analyses of variance (PERMANOVA). All subsequent statistical methods refer to the 454 gene tag pyrosequencing dataset.

Good's estimator, the Chao1 estimator and rarefaction analyses were conducted, as implemented in MOTHUR, to assess how well the recovered microbial communities represented the total microbial community. This analysis was performed after removing singleton OTUs (OTUs containing only one read). Specifically, these metrics provide estimates of total coverage, total expected OTU diversity and saturation of OTU accumulation (i.e., collector's) curves, respectively.

Microbial community similarity among sponge hosts was compared using both OTU-dependent (Bray-Curtis, BC; partial least squares discriminant analysis, PLS-DA) and OTU-independent calculations (UniFrac, Lozupone and Knight, 2005). BC indices were calculated based on the relative abundances of OTUs and visualized in cluster plots using MOTHUR. Significant differences in microbial community similarity were determined using PERMANOVA for the factors biogeography (Israel vs. Italy) and color morph (violet vs. white) and visualized in cluster plots. Factors exhibiting significant differences were further investigated by similarity percentage analyses (SIMPER) to identify microbial OTUs contributing to community dissimilarity. All PERMANOVA and SIMPER calculations were performed using Primer v6 and PERMANOVA+ (Plymouth Marine Laboratory, UK). PLS-DA was performed using the METAGENassist web server tool (Arndt et al., 2012) and the 97% OTU dataset. UniFrac analysis (Lozupone and Knight, 2005) was performed on rarefied data sets using the MOTHUR pipeline and cluster plots were constructed from the weighted UniFrac pairwise distance matrix, based on a phylogenetic tree constructed using the Clearcut module (Evans et al., 2006). PLS-DA loadings plot was analyzed and OTUs that mostly effected a separation were described.

### Chlorophyll *a* measurements

Chlorophyll *a* (chl *a*) concentrations were determined following the method described in Erwin et al. (2012a). Briefly, 0.25 g of ectosomal tissue (wet weight) from each sample was extracted overnight at 4°C with 5 ml of 90% acetone. Absorbance values of supernatant aliquots were determined at 750, 664, 647, and 630 nm and chl *a* concentrations were calculated using the equations of Parsons et al. (1984), standardized by sponge mass extracted. Chl *a* concentrations were compared between host sponge color morphs using One-Way analysis of variance (ANOVA) on ranked data (Kruskal-Wallis), since raw data deviated significantly from a normal distribution (Shapiro-Wilk, *P* < 0.05). Pairwise comparisons were conducted using the Student-Newman-Keuls (SNK) method. Statistical analyses were performed using the software SigmaPlot v11.

### Transmission electron microscopy (TEM)

Sponge pieces of ca. 4 mm^3^ from the violet (outside the cave), pink (cave entrance), and white (inside the cave) morphs of *P. ficiformis* were collected from a submarine cave located along the rocky cliffs of Paraggi (Portofino Promontory, Ligurian Sea, Italy). Each piece was fixed separately in a solution of 2.5% glutaraldehyde and 2% paraformaldehyde buffered with filtered seawater. Samples were incubated overnight at 4°C, then rinsed several times with filtered seawater and stored at 4°C until processed. Ultrathin sections were obtained with an Ultracut Reichert-Jung ultramicrotome, mounted on gold grids, and observed on a JEOL JEM-1010 (Tokyo, Japan) coupled with a Bioscan 972 camera (Gatan, Germany) for the acquisition of digital images. Ultrathin sections and TEM observations were performed at the Microscopy Unit of the Scientific and Technical Services of the University of Barcelona, Spain.

### Molecular identification of *P. ficiformis* sponges belonging to different color morphs

Sponges were identified by morphological analysis and sequencing of their 18S rRNA and cytochrome c oxidase subunit I (COX1) genes. A fragment of the 18S rRNA gene was amplified by PCR from Italian violet and pink *P. ficiformis* samples using primers: 18S_D1161b_PetrF (5′-TAGCGACTCCGTCGGCACCTCTC-3′) and 18S_PetrosiaV_1565_R (5′-AATCCTCCCTCGGCTAGAAAC-3′). The 18S rRNA gene from white *P. ficiformis* samples was amplified using the same forward primer, and the reverse primer 18S_PetrosiaW_1565_R (5′-AATCCTCCCTCGGCTTAGAAACC-3′). The PCR conditions were as follows: 95°C for 5 min; 35 cycles of 95°C for 30 s, 56°C for 30 s, 72°C for 30 s; and a final extension at 72°C for 3 min. A 222 bp alignment was used to build a maximum likelihood tree in MEGA 5.2. (Tamura et al., 2011), based on distance estimates calculated by the Kimura 2-parameter substitution model with gamma distributed rates among sites. This model was predicted as the best model for the current phylogenetic analysis by the Mega 5.2 best model prediction tool. Phylogenetic robustness was inferred from 1000 bootstrap replications.

The primers for amplifying the mitochondrial COX1 were LCO1490 (Folmer et al., 1994) and COX1-R1 (Rot et al., 2006). The conditions of PCR amplifications were: 95°C for 5 min; 35 cycles of 95°C for 40 s, 50°C for 50 s, 72°C for 1 min 30 s; and a final extension at 72°C for 10 min. The PCR products were gel purified on a 1% agarose gel and then extracted using the Promega Wizard® SV Gel and PCR Clean-UpSystem. A Maximum Likelihood tree based on distance estimates calculated by the Jones-Taylor-Thornton model with empirical frequencies was constructed in MEGA 5.2. This model was predicted as the best model for the current phylogenetic analysis by the Mega 5.2 best model prediction tool. Phylogenetic robustness was inferred from 1000 bootstrap replications.

Both PCRs were performed using 5× Red load Taq master (Larova) in a 50-μl PCR mixture with primer concentrations of 0.2 μM. For sequence alignments, additional 18S rRNA and COX1 genes sequences were downloaded from the NCBI nucleotide collection non-redundant database (http://www.ncbi.nlm.nih.gov/). Sequences were aligned using ClustalW (Thompson et al., 1994) as available in MEGA 5.2. 18S rRNA and COX1 amplicons were sequenced at Macrogen Europe (1105 AZ, Amsterdam, Netherlands).

## Results

### Microbial diversity and core community

A total of 40,507 16S rRNA sequences were obtained by 454 amplicon sequencing. These sequences were assigned to 1055 OTUs at the species level (distance 0.03), after removing singletons (Table S1). Rarefaction curves began to reach asymptotes and coverage estimates were high (>96%) for all samples, with the exception of sample PW3. Only 197 reads forming 94 OTUs were counted for this sample, with an estimated 181 OTUs (Chao1) predicted and 66.3% coverage. Insufficient low depth of sequencing of this sample led us to remove it from further analyses.

**Figure 2:**
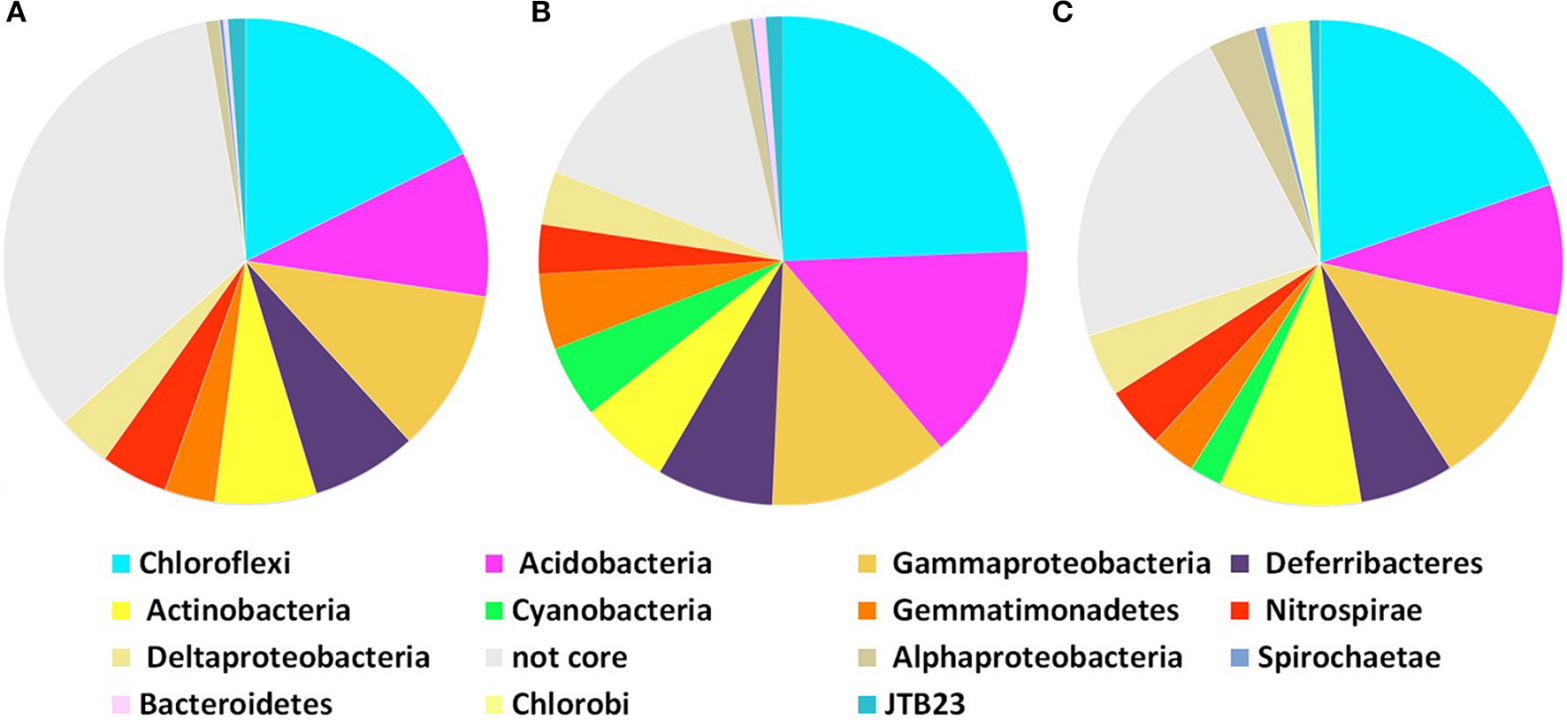
**Core community composition of microbial symbionts in *P. ficiformis* based on phylogenetic distribution of 97% OTUs at the phylum level (Proteobacteria are shown at the class level)**. Analysis does not include the experimental transplant specimens (D, Dt). Core symbiont communities in **(A)** all *P. ficiformis* samples, **(B)** violet *P. ficiformis* from Israel, and **(C)** violet *P. ficiformis* from Italy.

**Figure 3:**
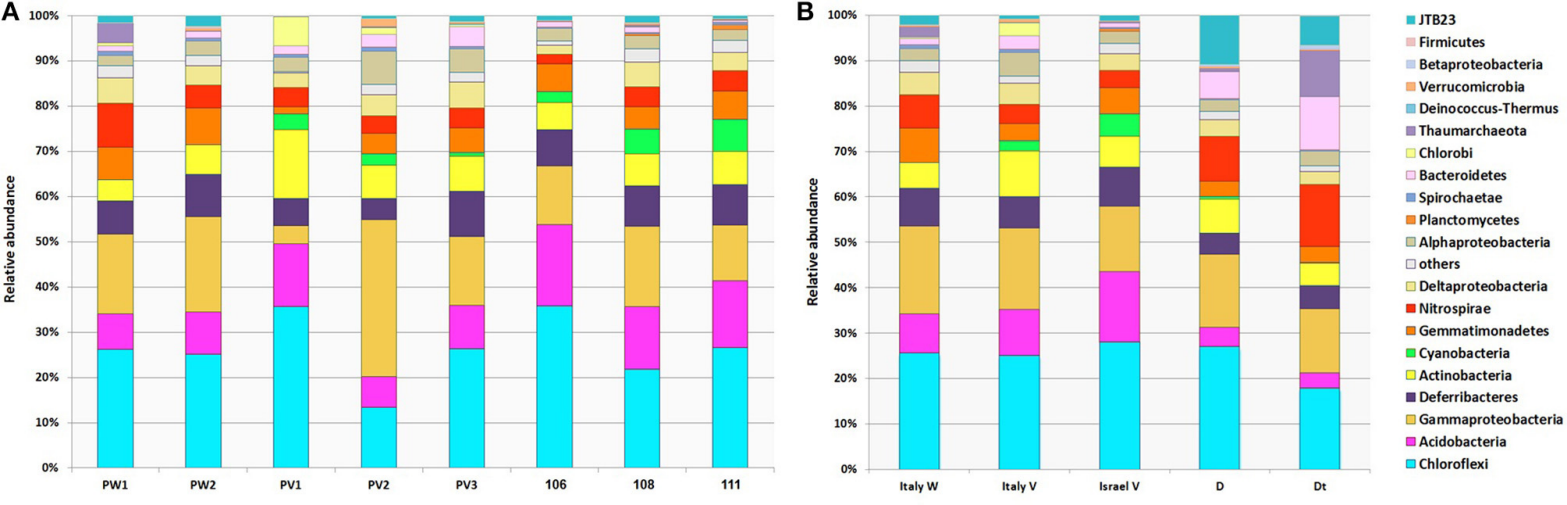
**Phylum-level composition of microbial symbionts in *P. ficiformis* among different color morphs and collection locations**. Distribution and relative abundances of OTUs from different phyla are shown for **(A)** each *P. ficiformis* specimen, and **(B)** grouped by different *P. ficiformis* morphs and collection locations. White morphs from Italy (PW1, PW2; Italy W), violet morphs from both Italy (PV1, PV2, PV3; Italy V) and Israel (106, 108, 111, Israel V) and an additional violet specimen, half transplanted to a dark cave for 6 months (Dt) and the other half kept at its original location for control (D) are shown. Relative abundances shown in this figure were constructed using OTUs with distance of 0.03 at the phylum level (Proteobacteria are shown at the class level). Groups are shown in the order in which they are listed.

Core microbial communities were defined as OTUs that appeared in all replicates of: (i) all *P. ficiformis* samples (70 OTUs), (ii) violet *P. ficiformis* from Italy (105 OTUs), and (iii) violet *P. ficiformis* from Israel (110 OTUs) (Figure 2). Chloroflexi and Gammaproteobacteria dominated all *P. ficiformis* samples, regardless of color morph and collection location (Figure 3), composed primarily of the Chloroflexi lineages SAR202 and TK10 and the Gammaproteobacteria lineages KI89A, Chromatiales and Xanthomonadales. Other phyla with OTUs present in all *P. ficiformis* samples included Acidobacteria (9.7%), Deferribacteres (7.1%), Actinobacteria (6.8%), Nitrospirae (4.5%), Deltaproteobacteria (3.6%), Gemmatimonadetes (3.3%), JTB23 (1.2%), Alphaproteobacteria (1%), Spirochaetes (0.3%) and Bacteroidetes (0.3%) (Figure 2A). The phylum Chlorobi (Cytophagales, OPB56) was present in higher abundances in violet sponges from Italy (PV1-3, Figure 2C) and comprised three OTUs (35, 677, 753).

### Community structure analyses

**Figure 4:**
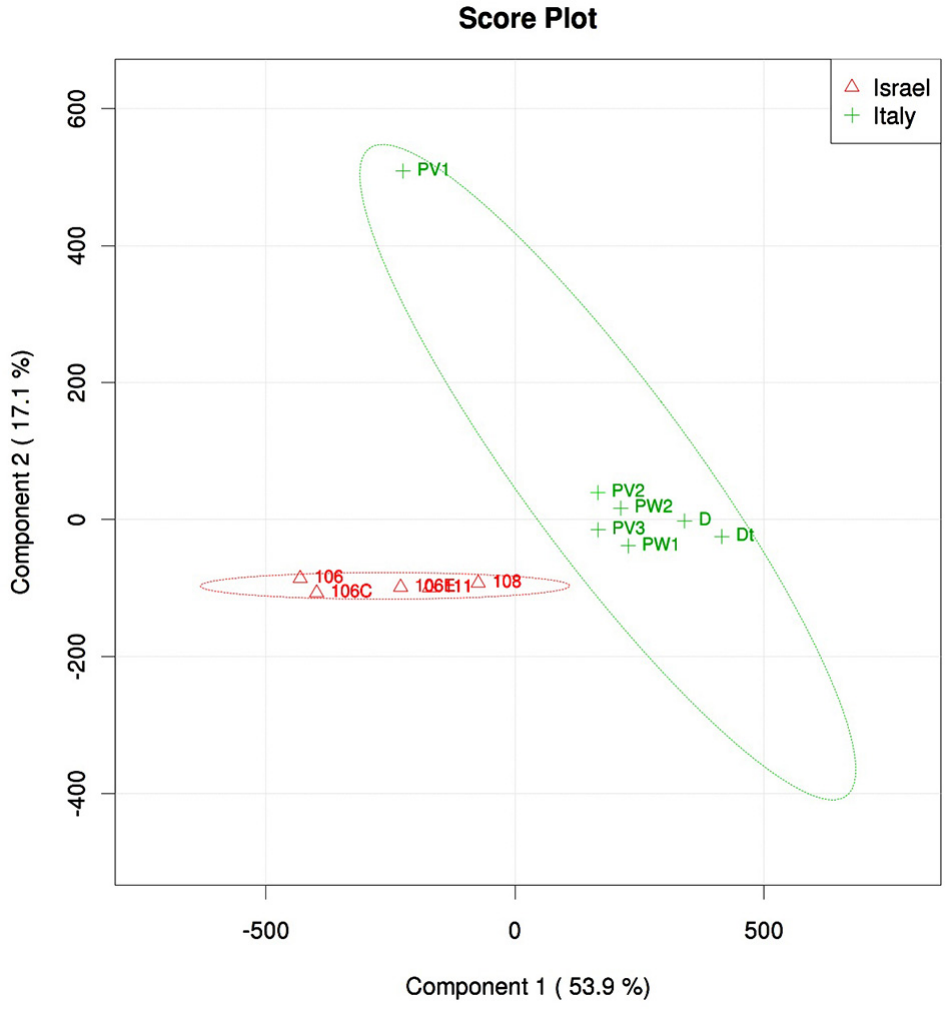
**PLS-DA score plot of microbial symbiont communities in *P. ficiformis* samples including aposymbiotic specimens (PW1, PW2, and Dt) and those hosting cyanobacteria (all the rest), and two geographic locations (red triangles for Israel and green cross for Italy)**. Table 1 lists OTUs that had the highest impact on the PLS-DA grouping.

Community structure analyses were performed using different methods, all showing a similar result: violet and white morphs of *P. ficiformis* had similar microbial communities (PERMANOVA, *P* = 0.18), while biogeography played a significant role in determining the community structure (*P* < 0.01, Table S2). The microbial communities associated with *P. ficiformis* from Italy exhibited differences from those associated with *P. ficiformis* from Israel, with the exception of sample PV1 (Figure 4). Table 1 (SIMPER) and Table S3 (PLS-DA) show the OTUs with the highest impact on the observed biogeographic grouping. OTUs 23, 33, 62, 69 belong to the SAR202 clade of Chloroflexi and were more abundant in Italian samples. OTUs 36 and 3 belonged to the Chloroflexi class Caldilineae and were more abundant in Israeli samples. OTU3 was also abundant in the Italian sample PV1. The Gammaproteobacteria OTUs 27 (KI89A clade) and 18 (E01-9C-26 marine group) were more abundant in Italian samples, while OTU43 (KI89A clade) showed higher abundance in Israeli samples. OTU45 (Marine group I, Thaumarchaeota) was unique to four Italian samples (D, Dt, PW1, and PW2), and its abundance was highly increased by translocation to the dark cave. OTU25 (Flavobacteria, Bacteroidetes) and OTU10 (JTB23, Proteobacteria) were prevalent in D and Dt. OTU6 (Nitrospira, Nitrospirae) was prevalent in Italian samples especially in D, Dt, and PW1. OTU13 (Acidobacteria, Acidobacteria) and OTU15 (PAUC34f, Deferribacteres) were prevalent in Israeli samples. OTU14 (Acidimicrobiales, Actinobacteria), OTU35 (Chlorobia, Chlorobi), and OTU19 (TK10, Chloroflexi) were prevalent in Italian sample PV1 and led to its separation on the PLS-DA ordination.

**Table 1:** **Similarity percentages (SIMPER) analysis showing the microbial OTUs that contributed most to the observed biogeographic trend in symbiont communities of *P. ficiformis***.

**OTU**	**Phylum (lowest taxon)**	**Average rel. abund.(%)**		**Contribution to dissimilarity**	
		**Israel**	**Italy**	**Individual**	**Cumulative**
3	Chloroflexi (Caldilineaceae)	13.19	2.83	9.42	9.42
8	Acidobacteria (Acidobacteria)	5.87	2.04	3.65	13.07
6	Nitrospirae (*Nitrospira*)	2.88	6.95	3.58	16.65
9	Cyanobacteria (*Synechococcus*)	4.44	0.97	3.43	20.07
13	Acidobacteria (Acidobacteria)	3.78	0.91	2.51	22.58
10	Proteobacteria (JTB23)	0.99	3.36	2.38	24.96

### Specificity and overlap of microbial OTUs

**Figure 5:**
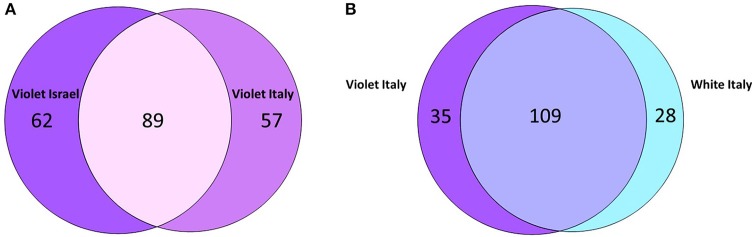
**Venn diagram comparing shared and unique 97% microbial OTUs in (A) violet *P. ficiformis* from Israel and Italy (*n* = 3 per group) and (B) violet (*n* = 3) and white (*n* = 2) *P. ficiformis* from Italy**.

Maintenance vs. variation in microbial OTUs among sampled *P. ficiformis* sponges was further investigated and quantified with Venn diagrams (Figure 5). An OTU was considered shared if it was present in at least two replicates of both groups that were compared. An OTU was considered unique if it was present in at least two replicates of one group and absent in all replicates of the other group. Violet *P. ficiformis* from Israel and Italy (*n* = 3 per group) had 89 shared OTUs, while the numbers of unique OTUs of violet *P. ficiformis* from Israel and Italy were 62 and 57, respectively. Consistent with the observed biogeographical trend, violet *P. ficiformis* (*n* = 3) and white *P. ficiformis* (*n* = 2) from Italy shared more OTUs (*n* = 109) than violet morphs from Israel and Italy (*n* = 89). The numbers of unique OTUs of violet and white *P. ficiformis* were 35 and 28, respectively.

### Chorophyll *a* concentrations and *Synechococcus feldmannii*

Chl *a* content differed significantly among color morphs (ANOVA, *P* < 0.01), with significant pairwise comparisons among violet (79.9 ± 31.5 μg/g), pink (12.9 ± 1.2 μg/g), and white (2.4 ± 1.4 μg/g) morphs (SNK, *P* < 0.05), confirming the presence of photosymbionts in violet and pink morphs of *P. ficiformis* and their absence in the white morph. Similarly, the 454 sequence dataset revealed the presence of the symbiotic cyanobacterium *S. feldmannii* in violet specimens, while it was absent from all white morphs of *P. ficiformis* (PW, Figures 2B,C). *S. feldmannii* (OTU9) was represented by 987 sequences and exhibited 99.5% similarity to previously described *S. feldmannii* from *P. ficiformis* (GenBank Acc. No. AY701297.1, Steindler et al., 2005). The transfer of half of the sponge D (violet *P. ficiformis*) to a cave caused the loss of coloration and disappearance of *S. feldmannii* (*S. feldmannii* represented 0.5% of the microbes in the violet specimen D and 0% in its bleached transferred half). When comparing cortex and endosome of specimen 106, *S. feldmannii* was present in the cortex (7.9%) and absent in the endosome (0%).

### Effect of photosymbionts on overall microbial communities

Given the presence of cyanobacterial symbionts in the cortex of *P. ficiformis* specimens growing in illuminated sites, we tested whether the presence of cyanobacteria affected the microbial communities found in the tissue hosting them. No significant differences were observed between microbial communities found in the cortex and endosome of violet *P. ficiformis* (PERMANOVA, *P* = 0.42). In addition, the transplantation of a violet morph of *P. ficiformis* from an illuminated site to a dark cave had little effect on the microbial symbiont community, despite apparent bleaching and loss of cyanobacterial symbionts (Figure 3), as the control and transplanted portions of this sponge were more similar to each other than sympatric violet and white sponges (Bray-Curtis analysis, data not shown).

One major effect observed in the transplant experiment was a noticeable increase in Thaumarchaeota, Marine group I, *Candidatus* Nitrosopumilus in the bleached part (control D = 0.8%, transplant Dt = 10.2%). There were three different Thaumarchaeota OTUs, with more than 10 reads in specimen Dt. The OTU responsible for most of the shift (0.8–10.2%) was OTU45 (Thaumarcheota, Marine Group I, *Candidatus* Nitrosopumilus).

### Ultrastructural observations (TEM)

**Figure 6:**
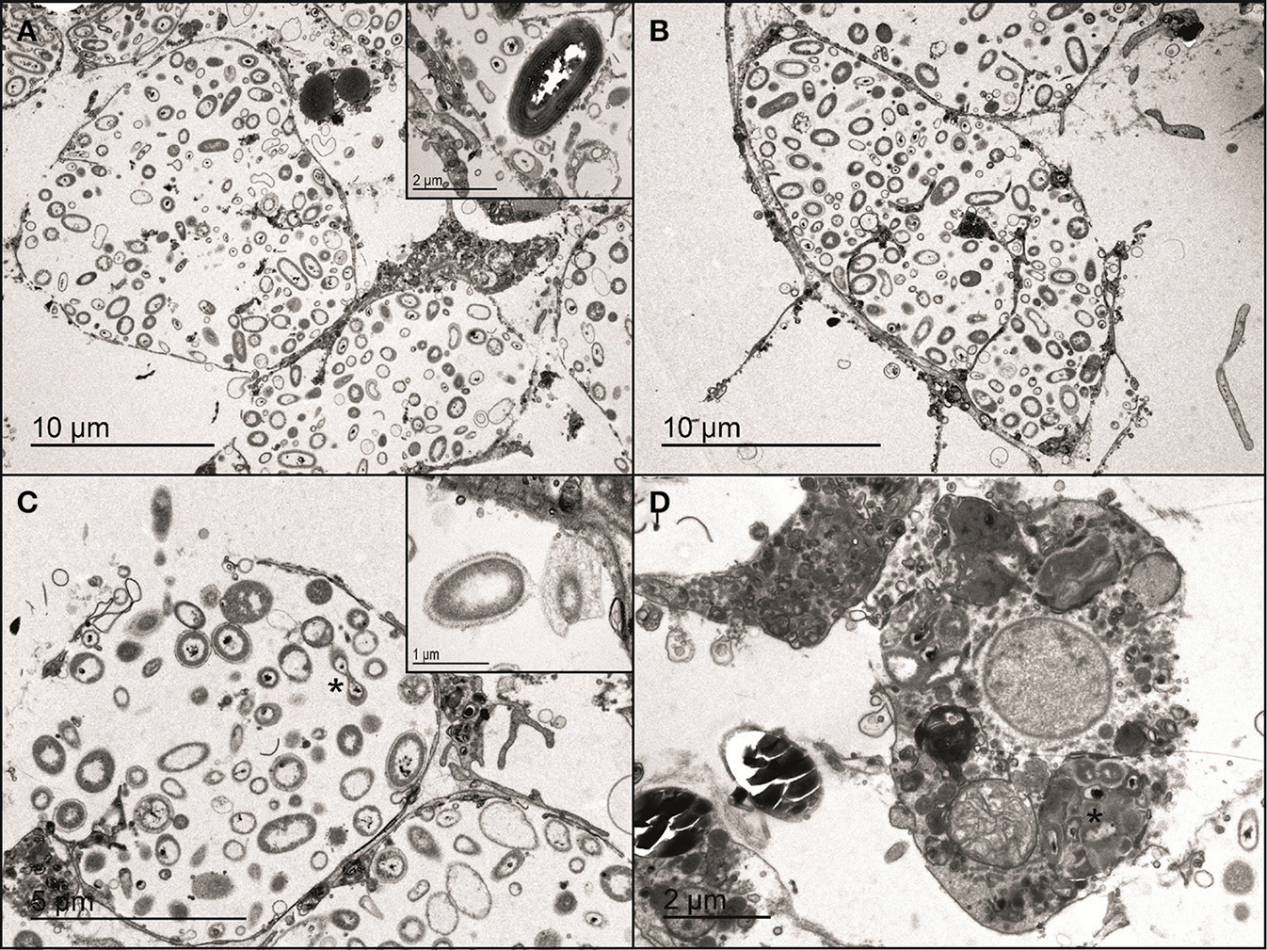
**Micrographs of violet, pink, and white morphs of the sponge *P. ficiformis*. (A)** Bacteriocyte in the mesohyl of the violet morph and **(B)** the white morph. The inset shows a cyanobacterial cell from the violet morph. **(C)** Dividing bacteria (asterisk) in a bacteriocyte of the violet morph. The inset shows bacteria interchanging cellular material in the white morph. **(D)** Phagocytic sponge cell containing a partially-digested cluster of bacteria (asterisk) in the pink morph.

Micrographs of the three color morphs (violet, pink, and white) of *P. ficiformis* showed numerous bacteriocytes in the mesohyl (Figures 6A,B). Each bacteriocyte contained a high diversity of bacteria, some of which were actively dividing (Figure 6C) or interchanging cellular material (Figure 6C). Bacteria were also observed surrounding the bacteriocytes or being actively phagocytized by sponge cells (Figure 6D). As expected, cyanobacterial cells were only observed in the violet and pink morphs of *P. ficiformis* and were absent in the white form (Figure 6A). Besides the presence/absence of cyanobacteria, no other differences in sponge or bacterial cell abundance and morphology were detected for any of the sponge morphs.

### Host phylogenetics

**Figure 7:**
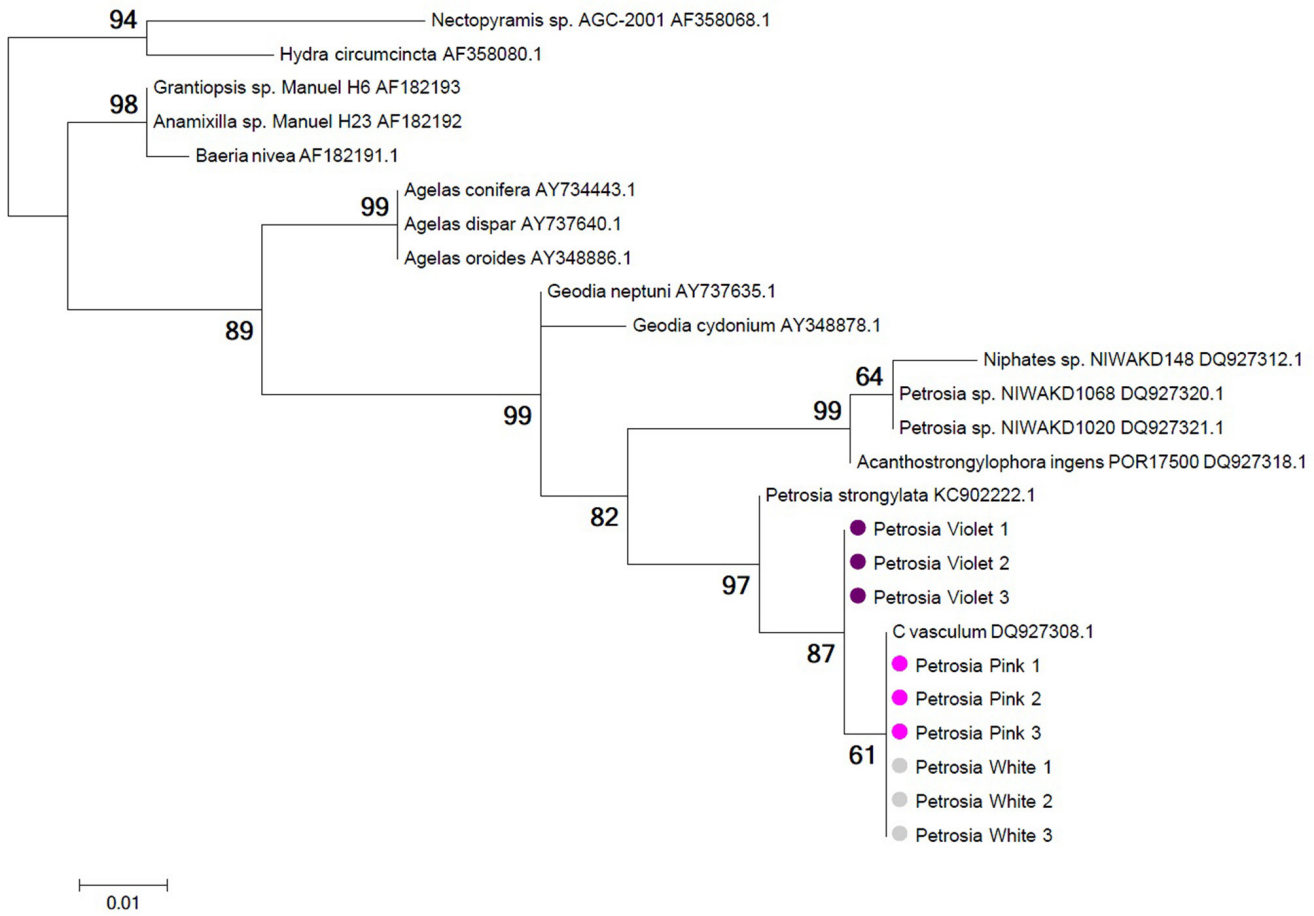
**Phylogeny of 18S rRNA gene sequences from white (gray circles), pink (pink circles), and violet (violet circles) morphs of *P. ficiformis***. Information on the geographic origin of the sponge specimens represented by the 18S rRNA sequences used in this tree is provided in Table S4 of Supplementary Material. Tree topology was constructed using maximum likelihood criteria and distance estimates calculated by the Kimura 2-parameter substitution model with gamma distributed rates among sites. Bootstrap values derive from 1000 replications and are shown at branch nodes. Tree construction was performed with MEGA5.2.

18S rRNA and mitochondrial COX1 genes amplified from violet, pink and white specimens in triplicates exhibited consistent differences between *P. ficiformis* color morphs. Maximum likelihood phylogenies showed a more derived and genetically-distinct cluster of *P. ficiformis* comprised solely of pink/white morphs for both genes, with a bootstrap support >60% in both analyses (Figure 7 and data not shown). The difference in COX1 gene sequences between the pink/white clade and the group formed by specimens from the violet morph was 3 nucleotides that resulted in variation of one amino acid (data not shown).

## Discussion

In this study, we investigated the structure of symbiotic microbial communities in *P. ficiformis* among color morphs and biogeographic locations, using a suite of molecular, chemical and microscopy analyses. Chlorophyll *a* content, TEM observations and DNA sequence data all confirmed the presence of cyanobacteria, specifically *S. feldmannii*, in violet and pink morphs of *P. ficiformis* and their absence in white morphs. Contrary to expectations, the presence/absence of these photosymbionts had little effect on overall microbial community composition, as evidenced through the comparison of white and violet morphs, the transplantation of a violet morph from an illuminated site to a dark cave habitat, and the comparison of cortex vs. endosome tissue from violet morphs. A previous study on various sponge species, including *P. ficiformis*, described high dissimilarities between microbial communities from cortex and endosome of high microbial abundance sponges. However, *P. ficiformis*, among others, was the species showing the least dissimilarity between tissue types (Gerce et al., 2011). The latter study analyzed a single specimen per analyzed sponge species, while in our investigation the comparison of cortex vs. endosome on triplicate samples did not show a grouping according to tissue type.

Rather than cyanobacterial symbionts (i.e., color morphs) accounting for variability in microbial symbiont communities, a biogeographic trend was observed between *P. ficiformis* collected in Israel and Italy. Surprisingly, DNA sequencing of ribosomal and mitochondrial markers revealed two genetically distinct clades of *P. ficiformis*: one composed of violet morphs and one of pink and white morphs. Taken together, these data show that microbial symbiont communities are more similar in genetically distinct *P. ficiformis* from the same location, than genetically similar *P. ficiformis* from distant locations. The difference in the sampling time of Italian and Israel samples (August and February for Italian and May for Israel specimens, respectively) was likely not the cause of the observed biogeographic effect, given that Italian samples from August and February grouped together (e.g., PW1 and PW2). In addition, temporal stability of sponge-associated microbial communities has recently been shown for other Mediterranean sponge species (e.g., Erwin et al., 2012b; Bjork et al., 2013; Hardoim and Costa, 2014).

Typical constituents of the sponge microbiome, previously described for many other sponge species (Schmitt et al., 2012b), were also found in *P. ficiformis*, with Chloroflexi (TK10, SAR202, Caldilinea, Anaerolineae; Schmitt et al., 2011; Bjork et al., 2013), Gammaproteobacteria (e.g., Xanthomonadales, Jackson et al., 2012) and Acidobacteria (e.g., PAUC26f, Hentschel et al., 2002) constituting approximately half of the microbiome. Additional typical sponge-specific clades included Actinobacteria (Sva0966 marine group) and Deferribacteres (e.g., PAUC34f, Moitinho-Silva et al., 2013). The main phyla found in the microbiome of *P. ficiformis* (Chloroflexi, Proteobacteria, and Acidobacteria) confirm results from a previous study (Schmitt et al., 2012a), however we found only few reads corresponding to Poribacteria, while in the sample of Schmitt et al. (2012a) Poribacteria represented 8% of the microbiome. A potential explanation for this discrepancy is that different primers (with different bias) were used in the two studies.

Most studies have shown high stability of sponge-associated microbial communities within the same sponge species collected across wide geographic distances (e.g., Pita et al., 2013a,b), though some exceptions have also been documented (Taylor et al., 2005; Lee et al., 2009b; Anderson et al., 2010). In the present study, we show that microbial communities in *P. ficiformis* are structured primarily by geographic location rather than host morph or presence/absence of cyanobacterial symbionts. Only one sample (PV1) did not conform to this biogeographic trend, instead it appeared to differ from all other samples, but to be closer to Israeli samples than Italian ones. This sample froze during storage (likely due to leakage of ethanol from the vial during transfer from Italy to Israel). Freeze-and-thaw of this sample may have biased the DNA extraction of specific bacterial groups resulting in the observed microbial diversity found for this sample. However, the alternative explanation, that a different general pattern may be observed if a larger sample size was to be inspected, cannot be excluded at this stage. The stability of microbial communities across distant locations found for other sponge species may be related to the location of symbiont within the host and the type of symbiont transmission utilized by the host. In most sponges, symbionts are found extracellularly within the mesohyl matrix and many bacteria are transferred to the next host generation through vertical transmission (i.e., transfer of symbionts via reproductive host cells: eggs, sperm, (e.g., Usher et al., 2001; Schmitt et al., 2008; Lee et al., 2009a; Gloeckner et al., 2013). In both these respects, *P. ficiformis* is quite an exceptional sponge: most bacteria are found intra-cellularly in bacteriocytes and embryos and larvae were found to be devoid of microbes (Maldonado, 2007). These characteristics may contribute to the biogeographic trends observed for microbial communities in *P. ficiformis* from Italy and Israel, with ambient bacterioplankton composition dictating which symbionts are available for colonization.

Before next generation sequencing (NGS) techniques were developed, it was suggested that sponge-specific bacteria may not be present in seawater and be only transferred vertically to the next sponge generation. NGS techniques revealed that sponge-specific bacteria are present in the seawater, though at very low concentration (Webster et al., 2010; Taylor et al., 2013). The fact that sponge-specific bacteria are found in *P. ficiformis* suggests that a yet undescribed recognition mechanism enables the acquisition of specific bacterial species from the ambient rare biosphere by sponges.

The greater influence of biogeography rather than association with cyanobacteria as structuring factor for symbiotic microbial communities in *P. ficiformis* contrasts our original hypothesis that photosymbionts (providers of an additional source of organic carbon) represent a keystone species in sponges. A possible explanation relates to the theory of functional equivalence between sponge microbiomes suggested by Fan et al. (2012). *P. ficiformis* also harbors ammonia-oxidizing archaea (*Nitrosopumilus* sp.) and ammonia-oxidizing bacteria (*Nitrosococcus*), thus the loss of carbon fixation processes by *S. feldmannii* may be compensated by a gain of chemoautotrophs. In fact, the transplant experiment revealed a major increase in the relative abundance of the *Nitrosopumilus* sp. (0.8–10.2%), an archaeon with ammonia-oxidizing capabilities. However, these remain hypotheses that require further replication of transplant experiments and physiological measurements to be appropriately tested.

The present study also provided molecular evidence for early speciation between the violet and the pink/white morphs of *P. ficiformis*. 18S rRNA and COX1 analyses showed that all samples from the violet morph of *P. ficiformis* (from Italy and Israel) grouped together and were basal to a clade formed by all the pink and white specimens. Notably, molecular evidence retrieved in this study supports previous morphological observations that described two distinct morphs of *P. ficiformis*: violet, tabular-shaped morphs on vertical relief habitats, and pink or white, cylindrical-shaped morphs growing inside or near the entrance of submarine caves (Vacelet and Donadey, 1977; Sarà et al., 1998). The molecular analyses presented herein also indicate that the pink-white, cave-associated morph is more derived, suggesting an earlier colonization of cave habitats by violet specimens, followed by subsequent diversification of the *P. ficiformis* species complex in cave habitats. This new condition seems to preclude the re-colonization of the lighted habitats by the sponge larvae coming from caves as documented by necrosis of ectosome in case of transplant of adult *P. ficiformis* from inside the cave to the outside (Regoli et al., 2000). Environmental factors potentially involved in this speciation process include the different hydrodynamic conditions found externally to the cave vs. at its entrance and inside the cave. The slender and cylindrical morphology with higher pore numbers observed in cave specimens may have resulted from low hydrodynamic conditions where higher metabolic rates and filtration capacities are required to match the carbon requisites of the holobiont. While additional molecular, morphological and reproductive data are needed to confirm this interesting finding, the similarity in microbial communities among morphotypes of *P. ficifiormis* and their divergence across locations provide new insights into the factors that structure symbiont communities in marine sponges.

### Data deposition

All sequences have been deposited in GenBank, accession numbers KM452895-KM452903 (18S rRNA sequences) and KM452904-KM452912 (COX1 gene sequences). The raw 454 amplicon pyrosequencing data were deposited in the NCBI Sequence Read Archive under project number PRJNA259436. NCBI Biosamples and SRA experiments accession numbers are given in Table S5.

### Conflict of interest statement

The authors declare that the research was conducted in the absence of any commercial or financial relationships that could be construed as a potential conflict of interest.
