# Supplementary material for: Biogeography rather than association with cyanobacteria structures symbiotic microbial communities in the marine sponge Petrosia ficiformis
Source: Front Microbiol. 2014 Oct 10;5:529. doi: 10.3389/fmicb.2014.00529 (PMC4193313; doi:10.3389/fmicb.2014.00529)
Supplement: Supplementary file 1 [file Table1.PDF]

Table S1. Samples with total reads, total OTUs, Chao1 estimate and coverage.

**Sample**	**106**	**106C**	**106E**	**108**	**111**	**D**	**Dt**	**PV1**	**PV2**	**PV3**	**PW1**	**PW2**	**PW3**
**Number of sequences**	3325	3309	3036	3077	3187	3302	3586	4707	3315	3113	2592	3761	197
**Number of OTUs**	222	231	229	321	293	278	266	217	291	300	280	334	94
**Predicted OTUs (CHAO1)**	286	324	282	398	348	327	324	272	356	359	335	414	181
**Coverage (%)**	97.7	97.5	97.6	96.3	97.2	97.5	97.7	98.6	97.2	97.2	96.7	97.1	66.3
