# Supplementary material for: Biogeography rather than association with cyanobacteria structures symbiotic microbial communities in the marine sponge Petrosia ficiformis
Source: Front Microbiol. 2014 Oct 10;5:529. doi: 10.3389/fmicb.2014.00529 (PMC4193313; doi:10.3389/fmicb.2014.00529)
Supplement: Supplementary file 2 [file Table2.PDF]

Table S2. Permutational multivariate analyses of variance (PERMANOVA) based on Bray-Curtis similarity of microbial communities in *P. ficiformis* (454 dataset). Results are shown for the factors collection location (Israel vs. Italy) and color morph (violet vs. white).

**Factor**	**df**	***MS***	***Pseudo-F***	***P (perm)***	***CV (%)***
Collection Location	1	3177.5	3.021	0.006	29.05
Color morph	1	1451.3	2.253	0.177	7.08
Residual	9	1051.7	-	-	63.87

df = degrees of freedom
P (perm) = P-values based on 999 permutations
CV = Component of Variation (square root)
