# Supplementary material for: Biogeography rather than association with cyanobacteria structures symbiotic microbial communities in the marine sponge Petrosia ficiformis
Source: Front Microbiol. 2014 Oct 10;5:529. doi: 10.3389/fmicb.2014.00529 (PMC4193313; doi:10.3389/fmicb.2014.00529)
Supplement: Supplementary file 3 [file Table3.PDF]

Table S3. Biogeographic separation of samples observed in the PLS-DA score plot resulted from presence and abundance of specific OTUs. A detachment character was evident for sample PV1, thus wherever 'Italy' is written as prevailing among samples, we intend 'Italy except sample PV1'. ND – not determined

OTU # (97%)	Prevailing among samples	Phylum	Class	Order/Clade/Group	Genus
**OTU 6**	Italy	Nitrospirae	Nitrospira	Nitrospirales	Nitrospira
**OTU 10**	Italy	Proteobacteria	JTB23	ND	ND
**OTU 25**	Italy	Bacteroidetes	Flavobacteria	Flavobacteriales	Owenweeksia
**OTU 45**	Italy	Thaumarchaeota	Marine Group I	Order *incertae sedis*	Candidatus Nitrosopumilus
**OTU 27**	Italy	Proteobacteria	Gammaproteobacteria	KI89A clade	ND
**OTU 18**	Italy	Proteobacteria	Gammaproteobacteria	E01-9C-26 marine group	ND
**OTU 23, 33, 62, 69**	Italy	Chloroflexi	SAR202 clade	ND	ND
**OTU 3**	Israel	Chloroflexi	Caldilineae	Caldilineales	Caldilinea
**OTU 13**	Israel	Acidobacteria	Acidobacteria	ND	ND
**OTU 15**	Israel	Deferribacteres	PAUC34f	ND	ND
**OTU 43**	Israel	Proteobacteria	Gammaproteobacteria	KI89A clade	ND
**OTU 36**	Israel	Chloroflexi	Caldilineae	Caldilineales	Caldilinea
**OTU 14**	PV1	Actinobacteria	Acidimicrobiia	Acidimicrobiales, Sva0996 marine group	ND
**OTU 35**	PV1	Chlorobi	Chlorobia	Chlorobiales	Cytophagales/ OPB56
**OTU 19**	PV1	Chloroflexi	TK10	ND	ND
