# Supplementary material for: Biogeography rather than association with cyanobacteria structures symbiotic microbial communities in the marine sponge Petrosia ficiformis
Source: Front Microbiol. 2014 Oct 10;5:529. doi: 10.3389/fmicb.2014.00529 (PMC4193313; doi:10.3389/fmicb.2014.00529)
Supplement: Supplementary file 4 [file Table4.PDF]

Table S4. List of specimens used in the phylogenetic analysis shown in Figure 7, including geographical origin, GenBank accession numbers of the 18S rRNA sequences and authors.

Classification	Origin	Accession number	Author
**Phylum Porifera**			
**Class Calcarea**			
Anamixilla sp.	GenBank	AF182192	Direct submission
Baeria nivea	GenBank	AF182191	Borchiellini et al. (2001)
Grantiopsis sp.	GenBank	AF182193	Direct submission
**Class Demospongiae**			
**Order Agelasida**			
Agelas conifera	GenBank	AY734443	Direct submission
Agelas dispar	GenBank	AY737640	Direct submission
Agelas oroides	GenBank	AY348886	Borchiellini et al. (2004)
**Order Astrophorida**			
Geodia cydonium	GenBank	AY348878	Borchiellini et al. (2004)
Geodia neptuni	GenBank	AY737635	Direct submission
**Order Haplosclerida**			
**Suborder Haplosclerina**			
Cribochalina vasculum	Bahamas	DQ927308	Redmond et al. (2007)
Niphates sp.	Micronesia	DQ927312	Redmond et al. (2007)
**Suborder Petrosina**			
Acanthostrongylophora ingens	Indonesia	DQ927318	Redmond et al. (2007)
Petrosia sp. NIWAKD1068	Papua New Guinea	DQ927320	Redmond et al. (2007)
Petrosia sp. NIWAKD1020	Papua New Guinea	DQ927321	Redmond et al. (2007)
Petrosia strongylata	Palau	KC902222	Redmond et al. (2013)
Petrosia ficiformis pink 1 (PP1)	Italy	KM452900	This study
Petrosia ficiformis pink 2 (PP2)	Italy	KM452899	This study
Petrosia ficiformis pink 3 (PP3)	Italy	KM452898	This study
Petrosia ficiformis violet 1 (PV1)	Italy	KM452903	This study
Petrosia ficiformis violet 2 (PV2)	Italy	KM452902	This study
Petrosia ficiformis violet 3 (PV3)	Italy	KM452901	This study
Petrosia ficiformis white 1 (PW1)	Italy	KM452897	This study
Petrosia ficiformis white 2 (PW2)	Italy	KM452896	This study
Petrosia ficiformis white 3 (PW3)	Italy	KM452895	This study
**Phylum Cnidaria**			
Hydra circumcincta	GenBank	AF358080	Medina et al. (2001)
Nectopyramis sp.	GenBank	AF358068	Medina et al. (2001)

References:

Borchiellini, C., Manuel, M., Alivon, E., Boury-Esnault, N., Vacelet, J. & Le Parco, Y. 2001. Sponge paraphyly and the origin of Metazoa. *J Evol Biol* 14, 171-79.

Borchiellini, C., Chombard, C., Manuel, M., Alivon, E., Vacelet, J. & Boury-Esnault, N. 2004. Molecular phylogeny of Demospongiae: implications for classification and scenarios of character evolution. *Mol Phylogenet Evol* 32, 823-37.

Redmond, N. E., van Soest, R. W. M., Kelly, M., Raleigh, J., Travers, S. a a & McCormack, G. P. 2007. Reassessment of the classification of the Order Haplosclerida (Class Demospongiae, Phylum Porifera) using 18S rRNA gene sequence data. *Mol Phylogenet Evol*, 43, 344–52.

Redmond, N. E., Morrow, C. C., Thacker, R. W., Diaz, M. C., Boury-Esnault, N., Cárdenas, P., et al. 2013. Phylogeny and systematics of demospongiae in light of new small-subunit ribosomal DNA (18S) sequences. *Integr Comp Biol*, 53, 388–415.

Medina, M., Collins, a G., Silberman, J. D., & Sogin, M. L. 2001. Evaluating hypotheses of basal animal phylogeny using complete sequences of large and small subunit rRNA. *J Evol Biol*, 98, 9707–12.
