# Supplementary material for: Biogeography rather than association with cyanobacteria structures symbiotic microbial communities in the marine sponge Petrosia ficiformis
Source: Front Microbiol. 2014 Oct 10;5:529. doi: 10.3389/fmicb.2014.00529 (PMC4193313; doi:10.3389/fmicb.2014.00529)
Supplement: Supplementary file 5 [file Table5.PDF]

Table S5. Accession numbers of the NCBI Biosamples and SRA experiments opened under project PRJNA259436.

Sample	Biosample (NCBI)	Experiment (SRA)
**106**	SAMN03009563	SRS692880
**106E**	SAMN03009580	SRS692884
**106C**	SAMN03009581	SRS692885
**108**	SAMN03009582	SRS692886
**111**	SAMN03009584	SRS692887
**D**	SAMN03009585	SRS692888
**Dt**	SAMN03009586	SRS692889
**PV1**	SAMN03009587	SRS692890
**PV2**	SAMN03009589	SRS692891
**PV3**	SAMN03009590	SRS692892
**PW1**	SAMN03009591	SRS692893
**PW2**	SAMN03009592	SRS692894
